# Aggressive Clinicopathological Course of Myeloma with t(3;16) (q21;q22) Cytogenetic Abnormality

**DOI:** 10.4274/tjh.galenos.2018.2018.0049

**Published:** 2019-02-07

**Authors:** Süreyya Bozkurt, Müfide Okay, İbrahim Haznedaroğlu

**Affiliations:** 1 İstinye University Faculty of Medicine, Department of Medical Biology, İstanbul, Turkey; 2 Hacettepe University Faculty of Medicine, Department of Internal Medicine, Division of Hematology, Ankara, Turkey

**Keywords:** Multiple myeloma, Rare translocations, Cytogenetic abnormality

## To the Editor,

Multiple myeloma (MM) is a heterogeneous disease and patients present with a wide variety of cytogenetic anomalies reflecting the nature of the disease [1]. The aim of this letter is to report a rare karyotypic abnormality with an aggressive clinical course of MM.

**Figure 1:**
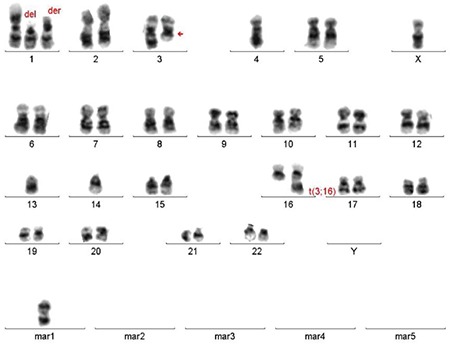
The patient’s karyotype.

A 56-year-old male patient was admitted to the neurosurgery clinic with dorsal shoulder pain and inability to walk in April 2011. He underwent thoracic and lumbar spinal magnetic resonance imaging. Laminectomy was performed on the patient upon detecting masses at the levels of the first and seventh thoracic vertebrae. The patient was referred to our center when he was determined to have “lymphoma” based on the first evaluation of his biopsy material. The specimen was then reevaluated in our center. A high-grade hematopoietic neoplasia was detected. Immunophenotypic findings suggested neoplasia with plasma cell origin. Immunohistochemically, neoplastic cells were positive for CD38, MUM-1, and kappa and negative for lambda. The karyotype of the patient was identified as 44,X,-Y,del(1)(p13p35),+der(1),t(3;16)(q21;q22),-4,-13,-14,+mar[8]/46,XY[42] (Figure 1).

Upon detection of newly developed lesions in the tenth thoracic vertebra in the control imaging obtained after the radiotherapy of the patient, treatment with vincristine, adriamycin, and dexamethasone (VAD) was initiated. The paraprotein levels in the patient’s serum decreased after four cycles of chemotherapy. However, the treatment was planned to be continued with only bortezomib (Velcade) and dexamethasone due to severe infection after chemotherapy and the development of decubitus infection during the follow-up of the patient. After three cycles of treatment, nodular lesions compatible with plasmacytomas appeared on his skin. The treatment strategy was changed since the patient was considered to have disease progression and had responded better to VAD chemotherapy. The patient was hospitalized due to pneumonia after chemotherapy and died due to severe sepsis.

The t(3;16)(q21;q22) anomaly reported in this case is a quite rare cytogenetic anomaly and, according to the databases that we have investigated [2,3], it was reported only in three adults and one child to date [4]. One of these three adult cases was a male patient with the diagnosis of myelodysplastic syndrome. In this patient, the translocation of t(3;16)(q21;q22) was found in the complex karyotype, as in our case [5]. The other two cases were female patients and they were diagnosed with acute myeloid leukemia that developed after acute myelomonocytic leukemia and primary tumor mantle cell lymphoma, respectively. None of the reported patients were diagnosed with MM. Therefore, we report the t(3;16)(q21;q22) anomaly, which is a quite rare translocation, in a patient with MM for the first time in this case. The prognostic effect of t(3;16)(q21;q22) is unknown. In the presented case, the anomaly of t(3;16)(q21;q22) coexisted with the anomalies of 1p deletion and monosomy 13, which are markers of progressive disease and short survival, or in other words, poor prognostic markers [6]. The clinical course of our case progressed in a manner that was consistent with poor prognosis. We believe that our case contributes to the literature since it is the first MM case with a very rarely encountered translocation.
